# Activated mammalian target of rapamycin is associated with T regulatory cell insufficiency in nasal polyps

**DOI:** 10.1186/1465-9921-10-13

**Published:** 2009-02-27

**Authors:** Geng Xu, Jiahong Xia, Xiaoyang Hua, Han Zhou, Chuanzhao Yu, Zheng Liu, Kemin Cai, Jianbo Shi, Huabin Li

**Affiliations:** 1 Allergy and Cancer Center, Otorhinolaryngology Hospital of the First Affiliated Hospital of Sun Yat-sen University, and Otorhinolaryngology Institute of Sun Yat-sen University, Guangzhou, PR China; 2 Department of Surgery, Tongji Medical College, Huazhong University of Science and Technology, Wuhan, PR China; 3 Department of Otolaryngology, the First Affiliated Hospital of Nanjing Medical University, Nanjing, PR China

## Abstract

**Background:**

Decreased infiltration of Foxp3+ T regulatory cell (Treg) is considered to be critical for the Th1/Th2 dysregulation of nasal polyps, while the cellular mechanism underlying Foxp3+ Treg insufficiency is currently not well defined.

**Methods:**

We attempted to investigate the tissue expression of phosphorylated mammalian target of rapamycin (pmTOR) and infiltration of Foxp3+ Tregs in 28 nasal polyps and 16 controls by histological staining. We also evaluated the effects of blocking the mTOR signaling pathway with rapamycin on T cell phenotype selection and Foxp3+CD4+ Tregs expansion in a tissue culture system.

**Results:**

Significantly increased infiltration of pmTOR+ inflammatory cells and decreased infiltration of Foxp3+CD4+ Tregs into nasal polyps was observed, with an inverse association. In the tissue culture system, we detected significantly elevated Foxp3 expression and IL-10 production, as well as an increased percentage of Foxp3+ Tregs in nasal polyps after blocking the mTOR signaling pathway with rapamycin.

**Conclusion:**

Here we demonstrate for the first time that the mTOR signaling pathway is associated with Foxp3+ Tregs insufficiency in nasal polyps. Inhibition of the mTOR signaling pathway may be helpful for enhancement of Foxp3+ Treg expansion, as well as modulation of T cell phenotype imbalances in nasal polyps.

## Background

Chronic rhinosinusitis is generally classified as chronic rhinosinusitis without nasal polyps (CRSnNP) or with nasal polyps (CRSwNP) [1]. CRSwNP is characterized by polyp formation and mixed types of Th1/Th2 infiltrates and their corresponding cytokine secretions [2,3]. There is also evidence that CRSwNP display a Th2-skewed inflammatory response with high levels of IL-5 and IgE [4]. At present, an imbalanced Th1/Th2 network is thought to play a critical role in the development of nasal polyps. However, the intercellular mechanisms underlying excessive T helper cell infiltration into nasal polyps have not been characterized.

Given the crucial role of T regulatory cell (Treg) in immune regulation, it is important to investigate their role in the pathogenesis of CRSwNP. Currently, at least two types of CD4+ Tregs have been partially characterized in humans: naturally occurring CD4+CD25+ Tregs and adaptive IL-10+/TGF-β+ CD4+ Tregs [5]. Naturally occurring CD4+CD25+ Tregs comprise a small proportion of CD4+ cells in mice and humans. The most specific biomarker of naturally occurring CD4+CD25+ Tregs is believed to be forkhead box P3 (Foxp3), a transcription factor that confers the regulatory phenotype to T cells [6]. There is increasing evidence that reduced Foxp3 gene expression or impaired Foxp3 function is potentially responsible for the development of autoimmunity and other diseases [7].

In our previous study, we observed that the expression of Foxp3 mRNA was downregulated in allergic rhinitis and nasal polyps [8,9], and treatment with a topical steroid enhanced the expression of Foxp3 mRNA and increased Treg accumulation in nasal polyps [10]. Similarly, Van Bruaene et al. recently demonstrated in a Western population with CRSwNP that decreased Foxp3 mRNA expression was accompanied by upregulated T-bet and GATA-3 mRNA, and downregulated TGF-β1 protein [11]. Together, these results provide evidences that decreased infiltration of Foxp3+ Tregs or Treg insufficiency is essential for dysregulation of the Th1/Th2 cytokine network in nasal polyps.

The potent ability of Foxp3+ Tregs to suppress immune responses has generated interest in harnessing their therapeutic potential to treat human diseases [7,12]. However, the signaling pathway underlying Foxp3+ Tregs expansion in humans has not been well characterized. Recent research has demonstrated that inhibition of the mammalian target of rapamycin (mTOR) is capable of fostering the selective survival and expansion of Foxp3+ Tregs [13]. mTOR is an evolutionarily conserved 289 kDa serine/threonine protein kinase that is inhibited by rapamycin [14]. In mammalian cells, mTOR integrates environmental cues such as nutrients, energy, and growth factors, and regulates cell growth and proliferation [15,16]. Most growth factors activate mTOR in a phosphoinositide-3-kinase (PI3K)-Akt-dependent fashion. In the presence of rapamycin, the PI3K-Akt-mTOR signaling pathway is inhibited, and multiple downstream targets of mTOR, such as 4E-BP1, are dysfunctional. We hypothesized that a hyper-activated mTOR signaling pathway contributes to Foxp3+ Treg insufficiency in nasal polyps. Therefore, the mTOR signaling pathway is a potential therapeutic target for Treg restoration.

To address this issue, we analyzed the protein expression of phosphorylated mTOR and Foxp3 in nasal polyps. We also evaluated the effects of rapamycin stimulation on the percentages of Foxp3+ Tregs and on the phosphatase and tensin homologue deleted on chromosome 10 (PTEN)/PI3K-Akt-mTOR signaling pathway in cultured nasal polyps. Our investigation may help to elucidate the pathogenesis of nasal polyps and provide an important strategy for modulating immune dysregulation by taking advantage of *in situ *Treg expansion in nasal polyps.

## Materials and methods

### Study subjects

Twenty-eight patients with CRSwNP were included in this study. Diagnosis of CRSwNP was based on clinical history, anterior rhinoscopy, nasal endoscopy, and paranasal CT scans. The patients met the criteria for CRSwNP according to the American Academy of Otolaryngology-Head and Neck Surgery Chronic Rhinosinusitis Task Force [1]. The presence of sinusitis or bilateral nasal polyps was confirmed by endoscopic inspection and CT scans. Polyps were graded according to the size and extent in both the left and right nasal fossa on a scale of 0 to 3. CT scans were graded by the Lund-Mackay staging system [17]. Atopic status was evaluated by a positive skin prick test (SPT) to at least one common inhalant allergens, including house dust mites, cat, dog, mixed cockroaches, and mixed molds. All patients had no history of asthma or other diseases. These CRSwNP patients were refractory to medical treatments (oral antibiotics, topical steroids, decongestants, and mucolytic agents for longer than six weeks), and had undergone endoscopic sinus surgery. Patients with a single polyp (antrochoanal, sphenochoanal) or with other diseases correlated with nasal polyps, such as cystic fibrosis, primary ciliary dyskinesia, and fungal rhinosinusitis, were excluded from the study. The use of local or systemic steroids or other medications was stopped at least four weeks before endoscopic sinus surgery. Sixteen patients with septum deviations were recruited as a control group. These subjects had no history of other respiratory pathology or allergy. They showed negative SPT to common inhalant allergens, such as house dust mites and mold. More detailed characteristics of the subjects are included in Table 1. This study was approved by the local institutional Ethics Committee and informed consent was obtained from all subjects.

**Table 1:** Characteristics of patients with CRSwNP and control subjects

Groups	CRSwNP	Control
Number	28	16
Sex(Male:Female)	16:12	8:8
Age(years)	37.7 ± 9.5(22~56)	34.7 ± 11.2(25~47)
Duration of disease (years)	3.4 ± 1.1(0.9~4.7)	NA
Skin prick test-positive	9/28	NA
Smoking	11/28	NA
Asthma in history and present	NA	NA
Aspirin intolerance	NA	NA
CT score (Lund-Mackay)	13.5 ± 2.7	NA
Total polyps scores	4.7 ± 1.1	NA

During surgery, polyp specimens and the inferior turbinate were sampled from CRSwNP patients and control subjects, respectively. Each specimen was divided into two portions. One portion was fixed in 4% paraformaldehyde and embedded in paraffin for further staining. The other portion was used immediately for nasal tissue culture.

### Immunohistochemistry

The immunoactivity of pmTOR was examined in all specimens using the avidin-biotin-peroxidase method according to our previous protocol. Briefly, paraffin sections (4 to 5 μm) were deparaffinized and rehydrated. Slides were incubated in 0.3% H_2_O_2 _for 10 min to eliminate the endogenous peroxidase. Specimens were heated for 10 min in 10 mM citrate buffer (pH 6.0) in a pressure cooker for epitope retrieval. Subsequently, the tissues were incubated with 10% bovine serum albumin for 1 h to block non-specific binding, followed by an overnight incubation at 4°C in the presence of a rabbit monoclonal antibody specific for pmTOR (Cell Signaling, Danvers, MA) at a dilution of 1:100. Each section was incubated with a secondary antibody (biotinylated goat anti-rabbit IgG, Zhongshan, Beijing, China), and then incubated with a horseradish peroxidase-labelled streptavidin complex (Zhongshan). The distribution of peroxidase was revealed by incubating the sections in a solution containing 3% 3, 3-diaminobenzidine tetrahydrochloride before counterstaining with hematoxylin. Negative control studies were performed by using isotype matched IgG or by omitting the incubation with the primary antibody according to the preliminary experiment, where isotype matched IgG experiment demonstrated no immunolabelling above background.

The sections were coded and analyzed under a light microscope with an eyepiece graticule. The number of pmTOR-positive (pmTOR+) cells in the epithelium and submucosae in 1 mm^2 ^of tissue was independently evaluated from ten reticules (10 × 0.1 mm^2^) randomly selected from a single section by two blinded investigators. A total of five sections per sample were examined.

### Double immunofluorescence staining

Cryostat sections (4 to 5 μm in thickness) were deparaffinized and rehydrated, blocked in 10% bovine serum albumin for 1 h, and incubated with primary antibodies overnight at 4°C. The primary antibodies included goat anti-human CD4 (1:100) and mouse anti-human Foxp3 (1:100). After rinsing, sections were incubated with secondary antibodies. First, a FITC-labelled donkey anti-goat antibody specific for CD4 (1:100) was incubated with the tissue sample for 1 h at room temperature. The Texas red-labelled donkey anti-mouse antibody for Foxp3 (1:100) was then incubated with the sample for 1 h at room temperature. After washing with PBS and nuclear staining with 4', 6-diamidino-2-phenylindole, dihydrochloride (DAPI, Santa Cruz), the slides were coverslipped with antifade reagent (Life Technologies, Rockville, MD). Negative control slides were prepared by omitting the primary antibody. Antibodies and DAPI were purchased from Santa Cruz Biotechnology, CA, U.S.A.

Nasal submucosal areas excluding glands below the basement membrane were viewed to quantify positive cells with an Olympus BX60 microscope (Olympus Optical Co, Japan) and the appropriate filter sets by two blinded investigators. Positive cells for CD4, Foxp3, and double-positive cells for CD4/Foxp3 were counted. Results were expressed as the number of positive cells and as the number of double-positive cells per mm^2^.

### Nasal tissue culture

Nasal tissue was rinsed three times with PBS containing antibiotics (50 IU/mL penicillin and 50 μg/mL streptomycin; Sigma-Aldrich, St.Louis, MO), and sectioned into multiple samples, as described elsewhere [18]. Tissue samples were weighed and each 100 mg section was cut into 1 to 2 mm^3^-large specimens, and placed in 1 ml of RPMI 1640 medium supplemented with 10% fetal calf serum (Life Technologies). The nasal tissues (control inferior turbinate, n = 16; nasal polyps, n = 28) were divided and either stimulated with 10 nM rapamycin (Sigma-Aldrich) or vehicle. All tissues were subsequently cultured at 37°C with 5% CO_2 _in humidified air for 48 h.

Supernatants and tissues were separated by centrifugation and collected for further study. All supernatants were analyzed by ELISA. Nasal tissues were randomly divided into two groups; some (control inferior turbinate, n = 9; nasal polyps, n = 15) were used for real time RT-PCR and immunoblot analyses, while others (control inferior turbinate, n = 7; nasal polyps, n = 13) were used for flow cytometric analysis.

### Real time reverse transcription polymerase chain reaction (RT-PCR)

Nasal tissues were collected by centrifugation and stored at -80°C until analysis. Real-time RT-PCR was performed as previously described [8]. RNA was extracted from nasal tissues using TRIzol reagent (Life Technologies) according to the manufacturer's instructions. Reverse transcription (RT) was performed, and cDNA was synthesized from 2 μg of total RNA using an oligo (dT)18 primer and M-MLV reverse transcriptase (TAKARA, Syuzou, Shiga, Japan). mRNA expression was determined using an ABI PRISM 7300 Detection System (Applied Biosystems, Foster City, CA) and SYBR Premix Taq™ (TAKARA). The sequences of the primers were as described elsewhere [19]: T-bet (NM_013351) forward: 5'-GAT GTT TGT GGA CGT GGT CTT G-3'; T-bet reverse: 5'-CTT TCC ACA CTG CAC CCA CTT-3'; GATA-3 (NM_002051) forward: 5'-GCG GGC TCT ATC ACA AAA TGA-3'; GATA-3 reverse: 5'-GCT CTC CTG GCT GCA GAC AGC-3'; Foxp3 (NM_014009) forward: 5'-GAG AAG CTG AGT GCC ATG CA-3'; Foxp3 reverse: 5'-AGG AGC CCT TGT CGG ATG AT-3' ; RORγt (NM_001001523) forward: 5'-TGA GAA GGA CAG GGA GCC AA-3'; RORγt reverse 5'-CCA CAG ATT TTG CAA GGG ATC A-3'; β-actin (NM_001101) forward: 5'-AAG ATG ACC CAG ATC ATG TTT GAG ACC-3'; β-actin reverse 5'-AGC CAG GTC CAG ACG CAG GAT-3'. PRISM samples contained 1 × SYBR Green Master Mix, 1.5 μL of 5 μM primers, and 25 ng of synthesized cDNA in a 25-μL volume. Reactions were heated to 95°C for 10 min, followed by 40 cycles of denaturation at 95°C for 10 s, and annealing extension at 60°C for 60 s. All PCR reactions were performed in duplicate. A melting curve analysis was used to control for amplification specificity. Routine PCR was performed and PCR products were analyzed with 1.5% agarose gel electrophoresis in the presence of ethidium bromide for UV light transilluminator visualization to confirm the expected size. The expression of the target gene was expressed as a fold increase or decrease relative to the expression of β-actin. The mean value of the replicates for each sample was calculated and expressed as a cycle threshold (Ct). The level of gene expression was calculated as the difference (ΔCt) between the Ct value of the target gene and the Ct value of β-actin. Fold changes of mRNA in nasal tissues were normalized to controls and determined as 2^-ΔΔCt ^.

### Flow Cytometric Analysis

Nasal tissues were collected after centrifugation, cut into small fragments, and teased apart to allow dispersion of the nasal cells into RPMI 1640. The cells were passed through a 40 μm mesh to obtain a single cell suspension. Following a rinse, cells were adjusted maximally to 2 × 10^6 ^cells/ml and labeled with the following anti-human mAbs: FITC conjugated CD4, APC conjugated CD25, and PE conjugated Foxp3 (eBioscience, San Diego, CA). Cell labelling was performed according to the manufacturer's instructions. Specifically, intracellular Foxp3 stain was labelled after prepared fixation/permeabilization buffer (eBioscience) was used. Cell fluorescence was measured using a FACSCalibur flow cytometer (BD Biosciences, San Diego, CA), and data were analyzed using CellQuest software (BD Biosciences).

### Western blotting

Nasal tissues were collected by centrifugation, lysed, and then stored at -80°C until analysis. The protein concentration was determined by the Bradford method. Samples containing 5 μg of protein were boiled and subjected to sodium dodecyl sulfate polyacrylamide gel electrophoresis in 10% Tris-glycine gels, and then transferred electrophoretically to a polyvinylidene fluoride membrane. The membrane was incubated with 5% non-fat milk in Tris buffered solution (TBS) containing 0.05% Tween 20 (1 h, room temperature) and incubated (overnight, 4°C) with anti-human monoclonal antibodies, including PTEN, PI3K, pAkt, pmTOR, p4E-BP1 (Cell Signaling) and T-bet, GATA-3, Foxp3, and β-actin (Santa Cruz), at different dilutions (1:1000 to 1:4000). The membrane was washed twice with TBS containing 0.05% Tween 20 and incubated with horseradish peroxidase-linked secondary antibodies (1:1000 to 1:4000). The immunoreactivity of proteins in the membrane was determined using an ECL chemiluminescence reaction kit, followed by exposure to medical film according to the manufacturer's instructions. The relative band density of the target protein to β-actin was quantified with Bio-Rad Quantity One 1-D Analysis Software (Bio-Rad, CA, USA).

### Enzyme-linked immunosorbent assay (ELISA)

The contents of cytokines in culture supernatants were determined by ELISA. The levels of IFN-γ, IL-4, IL-5, and IL-10 in the supernatants were determined using cytokine-specific ELISA kits (Bios, Beijing, China) according to the manufacturer's instructions. The sensitivity of the ELISA assay for cytokines was as follows: IFN-γ, 15.6 pg/ml; IL-4, 3.2 pg/ml; IL-5, 3.2 pg/ml; IL-10, 7.8 pg/ml. Assays were performed in duplicate. Results are expressed in pg/ml.

### Statistical analysis

Data are expressed as means ± SEM. The unpaired Student's *t *test for intergroup comparisons was applied for histologic examination and the quantitative relationship between the numbers of pmTOR+ inflammatory cells and Foxp3+CD4+ cells was assessed by linear regression. To evaluate tissue culture assay, a one-way ANOVA test and Bonferroni correction were applied for multiple comparisons, followed by a paired or unpaired Student's *t-*test for intragroup comparison. A *P *value less than 0.05 was considered as statistically significant.

## Results

### Increased pmTOR expression in nasal polyps by immunohistochemistry

**Figure 1:**
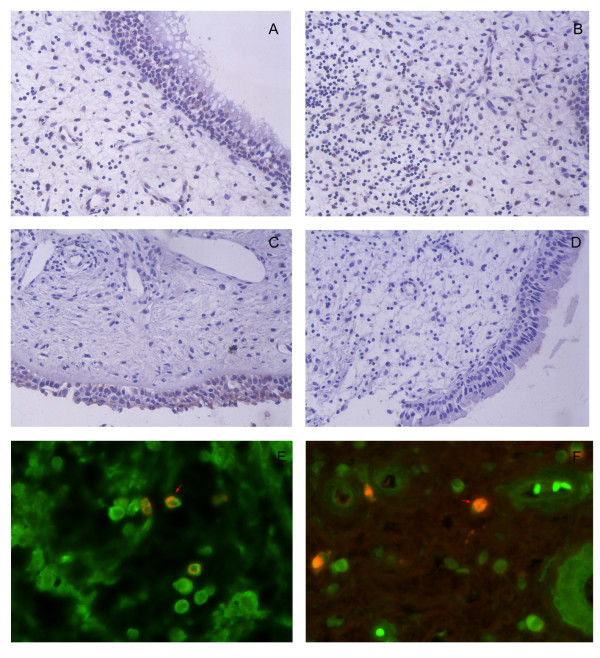
**Infiltration of pmTOR+ cells and Foxp3+CD4+ cell in nasal tissues by histologic examination**. The number of pmTOR+ cells was significantly higher in nasal polyps than in controls, whereas the number of Foxp3+CD4+ cells was significantly lower in nasal polyps than in controls. Representative images are provided and the results were analyzed by the unpaired *t*-test. (A), The pmTOR+ cells were located in both the epithelium and submucosa of nasal polyps; (B), Expression of pmTOR was primarily located in the submucosa of nasal polyps; (C) Immunostaining of pmTOR was localized primarily to the epithelium in control tissues; (D), Negative control for pmTOR staining in nasal polyps (200× magnification). (E and F), Foxp3+CD4+ cells were located in nasal polyps (E) and the control tissue (F), as evidenced by double immunofluorescence (400× magnification); CD4 FITC (green); Foxp3 Texas red (red); double-positive stain (arrow, orange).

In order to determine the phosphorylation status of mTOR in nasal tissues, pmTOR expression was examined by immunohistochemistry (Figure 1A, B, C, D) and pmTOR+ cells in the epithelium and submucosa (representing inflammatory cells) were quantified (Table 2). We found that cytoplasmic pmTOR immunostain was primarily located in subepithelial inflammatory cells. Additional stain was also detected in the pseudostratified ciliated columnar epithelium of the nasal mucosa. The number of pmTOR+ cells was significantly higher in the submucosa (*P *< 0.01 by unpaired *t*-test) and the epithelium of nasal polyps (*P *< 0.05 by unpaired *t*-test) compared to that in the control mucosa. Therefore, we provide evidence for significantly elevated infiltration of pmTOR+ cells into nasal polyps.

**Table 2:** Quantification of pmTOR+ cells and Foxp3+/CD4+ cells in nasal polyps (per mm^2^)

	Control tissue	Nasal polyps	
pmTOR+ cells in epithelium	258 ± 78	444 ± 207	*P *= 0.025
pmTOR+ cells in submucosa	145 ± 59	1063 ± 490	*P *= 0.001
CD4+ cells in submucosa	474 ± 269	535 ± 108	*P *= 0.062
Foxp3+ cells in submucosa	126 ± 65	49 ± 21	*P *= 0.037
Foxp3+CD4+ cells in submucosa	91 ± 44	41 ± 15	*P *= 0.015

Nasal polyps, n = 28; control inferior turbinate, n = 16

Data were expressed as means ± SEM. *P *value is determined compared by the unpaired *t*-test.

### Decreased Foxp3+CD4+ Tregs in nasal polyps by double immunofluorescence

In general, Foxp3 is thought to be a specific biomarker of naturally occurring Tregs, although it is also present in other non-CD4+ cells. In order to more precisely identify Foxp3+ Tregs in nasal polyps, we simultaneously evaluated CD4 and Foxp3 by double immunofluorescence staining. We observed that more than 80% of Foxp3 was located in CD4+ T cells based on overlaid double immunofluorescence stains. Representative slides of overlaid Foxp3+CD4+ cells in nasal tissues are shown in Figure 1E and Figure 1F. As shown in Table 2, the number of Foxp3+CD4+ cells decreased significantly in nasal polyps compared to control nasal tissues (*P *< 0.05 by the unpaired *t*-test). In order to evaluate whether Foxp3+CD4+ cells were associated with pmTOR+ inflammatory cells, we investigated the quantitative relationship between the numbers of pmTOR+ inflammatory cells and Foxp3+ CD4+ Tregs in nasal polyps by linear regression. Our results indicate that the number of Foxp3+CD4+ Tregs negatively correlated with the number of pmTOR+ inflammatory cells in nasal polyps (*b *= -0.74, *P *< 0.01).

### Rapamycin modulates the gene expression of T-bet, GATA-3, Foxp3, and RORγt in cultured nasal polyps

**Figure 2:**
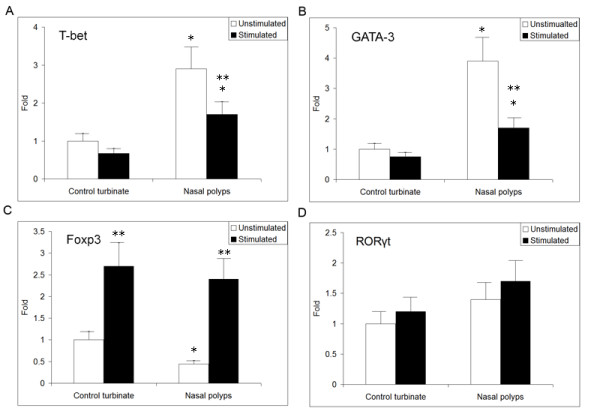
**Rapamycin modulates the relative levels of T-bet, GATA-3, Foxp3, and RORγt mRNA in cultured nasal polyps, as determined by real time RT-PCR**. Rapamycin stimulation has been shown to be associated with the expression of Foxp3 mRNA in nasal polyps. Elevated T-bet and GATA-3 mRNA and decreased Foxp3 mRNA were observed in nasal polyps (* *P *< 0.05 by the unpaired *t*-test for intergroup comparison) (A-C). After treatment with rapamycin, Foxp3 mRNA increased significantly in nasal polyps as well as in controls (5.5-fold and 2.7-fold, respectively) (**P < 0.05 by the paired *t*-test for intragroup comparison), whereas T-bet and GATA-3 mRNAs decreased significantly in nasal polyps (42% and 56%, respectively) (**P < 0.05 by paired *t*-test intra-group comparison). No significant changes in RORγt gene expression were found by multiple comparisons (D) (*P *> 0.0125 by the ANOVA test and Bonferroni correction).

Since the functional development of T cells is regulated by specific transcription factors, we quantified the levels of T-bet, GATA-3, Foxp3, and RORγt mRNA in rapamycin-stimulated nasal polyps by real time RT-PCR. As shown in Figure 2, the expression of T-bet, GATA-3, Foxp3, and RORγt mRNA was detected in all specimens, and significant changes in T-bet, GATA-3, and Foxp3 gene expression were observed during multiple comparisons (*P *< 0.0125 by the ANOVA test and Bonferroni correction). For intragroup comparison, we observed a significant elevation of T-bet and GATA-3 mRNA, but a significant reduction of Foxp3 mRNA in nasal polyps compared to the control mucosa (*P *< 0.05 by the unpaired *t*-test). After stimulation with rapamycin at a final concentration of 10 nM for 48 h, we found that Foxp3 mRNA was increased significantly in nasal polyps, as well as in the control (5.5-fold and 2.7-fold, respectively) (*P *< 0.05 by the paired *t*-test), whereas T-bet and GATA-3 mRNAs were significantly decreased in nasal polyps (42% and 56%, respectively) (*P *< 0.05 by the paired *t*-test). However, there was no significant change in RORγt gene expression during multiple comparisons (*P *> 0.0125 by the ANOVA test and Bonferroni correction). Therefore, our results provide evidence that rapamycin stimulation is associated with Foxp3 mRNA expression in nasal polyps.

### Rapamycin treatment is associated with an increase in Foxp3+ Tregs in cultured nasal polyps

**Figure 3:**
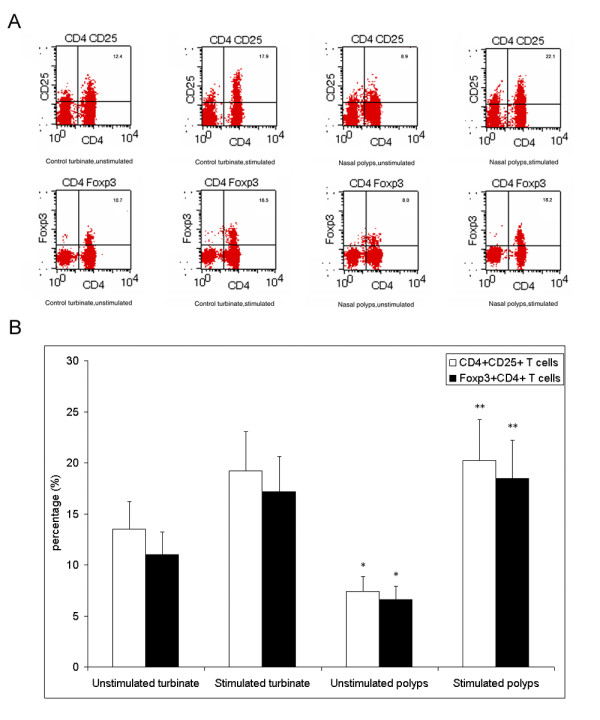
**Rapamycin increases the percentages of Tregs in cultured nasal polyps analyzed by flow cytometry**. Representative two-dimension scatter diagrams of CD4, CD25, and Foxp3 are shown (A) and we found that rapamycin treatment is associated with an increase in Foxp3+ Tregs in cultured nasal polyps(B). Significant changes in CD4+CD25+ and Foxp3+CD4+ cells were observed during multiple comparisons (*P *< 0.0125 by the ANOVA test and Bonferroni correction). The percentages of CD4+CD25+ and Foxp3+CD4+ cells significantly were decreased in nasal polyps, compared to the control (Figure 3) (*P < 0.05 by the unpaired *t*-test for intergroup comparison). After treatment with rapamycin, a significant increase in the frequencies of CD4+CD25+ cells and Foxp3+CD4+ Tregs was found in nasal polyps (**P < 0.05 by the paired *t*-test for intragroup comparison).

In order to evaluate the frequency of Foxp3+ Tregs in nasal polyps after rapamycin treatment, we examined CD4, CD25, and Foxp3 biomarkers in isolated cells from nasal polyps by flow cytometric analysis. Significant changes in CD4+CD25+ and Foxp3+CD4+ cells were observed by multiple comparisons (*P *< 0.0125 by ANOVA test and Bonferroni correction). For intragroup comparison, we found the percentages of CD4+CD25+ and Foxp3+CD4+ cells to be significantly decreased in nasal polyps compared to the control (Figure 3) (*P *< 0.05 by the unpaired *t*-test). After treatment with rapamycin, a significant increase in the frequencies of CD4+CD25+ cells and Foxp3+CD4+ Tregs in nasal polyps was found compared to untreated nasal polyps (*P *< 0.05 by the paired *t*-test). Thus, our results show that rapamycin administration is associated with elevated Foxp3+ Tregs in cultured nasal polyps

### Inhibition of mTOR signaling by rapamycin is associated with enhanced Foxp3 expression in cultured nasal polyps

**Figure 4:**
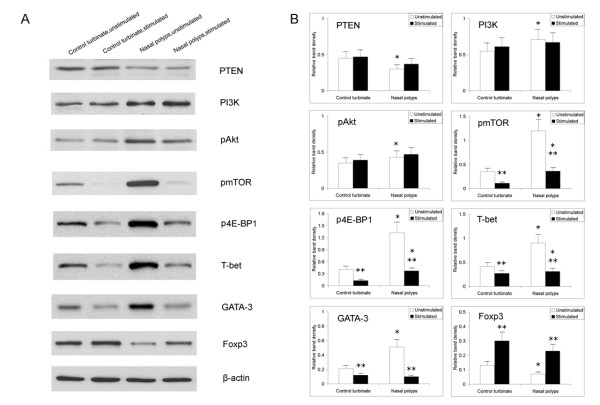
**Rapamycin modulates the protein levels of PTEN, PI3K, pAkt, pmTOR, p4E-BP1, T-bet, GATA-3, and Foxp3 in nasal polys**. Representative immunoblot data are shown (A) and significant changes in the levels of PTEN, PI3K, pAkt, pmTOR, p4E-BP1, T-bet, GATA-3, and Foxp3 were observed by multiple comparisons (*P *< 0.0125 by the ANOVA test and Bonferroni correction) (B). Significantly higher expression of PI3K, pAkt, pmTOR, and p4E-BP1 and lower expression of PTEN were observed in nasal polyps (* *P *< 0.05 by the unpaired *t*-test for intergroup comparison). After rapamycin stimulation, the protein levels of pmTOR and p4E-BP1 significantly were decreased in nasal polyps (70% and 72% for pmTOR and p4E-BP1, respectively; **P < 0.05 by the paired *t*-test for intra-group comparison), as well as in the control turbinate (69% and 68% for pmTOR and p4E-BP1, respectively; **P < 0.05 by the paired *t*-test for intragroup comparison). In contrast, the expression levels of PTEN, PI3K, and pAkt were not affected by rapamycin stimulation (*P *> 0.05 by the paired *t*-test for intragroup comparison). Furthermore, Foxp3 expression was significantly upregulated (3.3-fold in nasal polyps and 2.3-fold in control tissues; **P < 0.05 by the paired *t*-test for intra-group comparison) after rapamycin stimulation, while T-bet and GATA-3 expression correspondingly decreased (**P < 0.05 by the paired *t*-test for intra-group comparison).

Phosphorylation events in the PI3K-Akt-mTOR signaling pathway lead to the functional activity of downstream proteins, such as 4E-BP1. In order to evaluate the possible association between inhibition of mTOR signaling and Foxp3+ Treg expansion, we investigated a series of proteins, including PTEN, PI3K, pAkt, pmTOR, p4E-BP1, T-bet, GATA-3, and Foxp3 in rapamycin-stimulated nasal polyps by western blot analysis. Significant changes in the levels of PTEN, PI3K, pAkt, pmTOR, p4E-BP1, T-bet, GATA-3, and Foxp3 were observed by multiple comparisons (*P *< 0.0125 by the ANOVA test and Bonferroni correction). For intragroup comparison, significantly higher expressions of PI3K, pAkt, pmTOR, and p4E-BP1 and lower expression of PTEN were observed in nasal polyps compared to the control in the absence of rapamycin stimulation (*P *< 0.05 by the unpaired *t*-test) (Figure 4A, B). After rapamycin treatment, the protein levels of pmTOR and p4E-BP1 significantly decreased in nasal polyps (70% and 72% for pmTOR and p4E-BP1, respectively; *P *< 0.05 by the paired *t*-test), as well as in the control turbinate (69% and 68% for pmTOR and p4E-BP1, respectively; *P *< 0.05 by the paired *t*-test) compared to unstimulated tissues. In contrast, the expression of PTEN, PI3K, and pAkt were not affected by rapamycin stimulation (*P *> 0.05 by the paired *t*-test). Furthermore, Foxp3 expression was significantly upregulated (3.3-fold in nasal polyps and 2.3-fold in control tissues; *P *< 0.05 by the paired *t*-test) after rapamycin stimulation, while T-bet and GATA-3 expression decreased correspondingly (*P *< 0.05 by the paired *t*-test). These results are consistent with the histological findings, and confirm that mTOR signaling pathway is associated with Foxp3 expression and Foxp3+ Treg expansion in nasal polyps.

### Rapamycin modulates the levels of inflammatory cytokines in cultured nasal polyps

**Figure 5:**
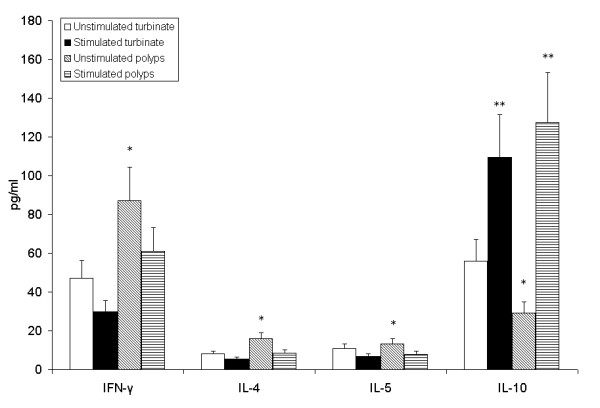
**Rapamycin modulates the levels of T cell cytokines in the supernatants of cultured nasal polyps, as determined by ELISA**. Significant changes were observed in the levels of IFN-γ, IL-4, IL-5, and IL-10 by multiple comparisons (*P *< 0.0125 by the ANOVA test and Bonferroni correction) and Rapamycin was shown to enhance IL-10 production in nasal polyps. More Th1/Th2 and less Treg cytokines were demonstrated in nasal polyps (*P < 0.05 by the unpaired *t*-test for intragroup comparison). After rapamycin treatment, a slight decrease in Th1 and Th2 cytokines, IFN-γ, IL-4, and IL-5 (30%, 47%, and 41%, respectively;**P > 0.05 by the paired *t*-test for intragroup comparison), and a significant increase in IL-10 were found in nasal polyps (4.4-fold; **P < 0.05 by the paired *t*-test for intragroup comparison).

Given that cytokine profiles reflect committed T cell phenotypes, we examined the contents of different cytokines (IFN-γ for Th1, IL-4 and IL-5 for Th2, and IL-10 for Tregs) in supernatants of the cultured nasal tissues by cytokine-specific ELISA and found significant changes in the levels of IFN-γ, IL-4, IL-5, and IL-10 during multiple comparisons (*P *< 0.0125 by the ANOVA test and Bonferroni correction). For intragroup comparison, more Th1/Th2 and less Treg cytokines was observed in polyps than in control (Figure 5) (*P *< 0.05 by the unpaired *t*-test), confirming that nasal polyps is characterized by mixed types of Th1/Th2 infiltrates and their corresponding cytokine secretions. After rapamycin treatment, a slight decrease in Th1 and Th2 cytokines, IFN-γ, IL-4, and IL-5 (30%, 47%, and 41%, respectively;*P *> 0.05 by the paired *t*-test), and a significant increase in IL-10 was found in nasal polyps compared to the untreated polyp tissues (4.4-fold; *P *< 0.05 by the paired *t*-test). These findings suggest that blocking mTOR singaling by rapamycin may enhance the function of Foxp3+ Tregs and IL-10 production in nasal polyps.

## Discussion

The choice of the Th1/Th2 lineage is important for effective immune responses to specific pathogens, while the balance between effector T cells and Tregs is vital for acquiring immune competence without immune pathology and autoimmunity [20]. In CRSwNP, the relationship between the Th1/Th2 lineage and Tregs is complex. Growing evidence suggests that nasal polyps display an imbalance between the Th1/Th2 lineage and Tregs. For example, several investigators have proposed that IFN-γ, IL-4, and IL-5-producing lymphocytes are found in nasal polyps, and that nasal polyps possess a mixed pattern of Th1/Th2 cytokines [2,21]. More recently, a Th2-skewed eosinophilic inflammation with high levels of IL-5 and IgE was described in CRSwNP, and decreased Foxp3 mRNA was demonstrated in nasal polyps. In our previous reports [8,9], we provided evidence that the level of Foxp3 protein and the number of Foxp3+ Tregs decreased significantly in nasal polyps. Given the critical role of Foxp3+ Tregs in immune tolerance, further investigation of the signaling pathways underlying Foxp3+ Treg enrichment in nasal polyps is of significant interest.

In the present study, we investigated the phosphorylation status of mTOR in a group of Chinese CRSwNP patients by immunohistochemistry, and the infiltration of Foxp3+CD4+ Tregs by double immunofluorescence staining. We observed significantly elevated infiltration of pmTOR+ cells in nasal polyps compared to controls. Moreover, the number of Foxp3+CD4+ cells was decreased significantly. Interestingly, we found the Treg insufficiency was negatively associated with the enhanced infiltration of pmTOR+ inflammatory cells. Therefore, our results raised the possibility that the mTOR signaling pathway may be associated with Foxp3+ Treg insufficiency in nasal polyps. To our knowledge, this is the first report examining mTOR signal in nasal polyps.

Since Foxp3+ Tregs comprise only a small proportion of the T cell population, it is important to investigate the mechanism involved in Treg expansion before attempting to harness their therapeutic potential for suppressing immune responses and treating human diseases. Recent studies have revealed that signaling pathways initiated by the T cell receptor, co-stimulatory molecules, IL-2 receptor, TGF- and beyond are closely associated with Foxp3 expression [22]. However, the physiological factors and pathways initiating intracellular Foxp3 expression and promoting its immune regulatory characteristics remain poorly understand. Research during the past two years has generated significant interest in the association of the PI3K/Akt/mTOR signaling pathway with Foxp3+ Treg differentiation [23,24]. For instance, rapamycin was capable of promoting Foxp3+CD4+CD25+ Tregs in T cell population in the blood [24,25]. In addition, antigen stimulation of naïve CD4 T cells in the presence of mTOR blockage by rapamycin favored their differentiation into the Foxp3+ phenotype, and these cells can participate in mediating antigen-specific tolerance [26,27]. These findings suggested that blocking the mTOR signal may be essential for the development of Tregs, which has potential significance in designing tolerance protocols to prevent graft rejection, treat autoimmunity, or enhance allergen-specific immunotherapy. However, little is known about the *in situ *expansion of Tregs in inflammatory tissues such as nasal polyps in response to blocking the mTOR pathway.

To determine the possible role of mTOR signaling in Foxp3+ Treg insufficiency in nasal polyps, we investigated the percentages of Foxp3+ Tregs and cytokine profiles in a tissue culture system after treatment with rapamycin. First, we have validated that the levels of pmTOR and p4E-BP1 were significantly decreased (by 70% and 72% for pmTOR and p4E-BP1, respectively) in rapamycin-treated nasal polyps, confirming that rapamycin functioned as a negative regulator of the mTOR pathway. Subsequently, we examined the levels of T-bet, GATA-3, Foxp3, and RORγt mRNA expression in cultured nasal polyps. These transcription factors are known to direct the commitment of four types of CD4+ T cells (T-bet for Th1, GATA-3 for Th2, Foxp3 for Tregs, and RORγt for Th17). Interestingly, our results, presented characteristically high levels of T-bet/GATA-3 mRNA and low levels of Foxp3 mRNA in nasal polyps, which is consistent with previous reports [2,3]. After treatment with rapamycin, we found that the level of Foxp3 mRNA was increased by as much as 5.5-fold in nasal polyps, while the levels of T-bet and GATA-3 mRNA decreased, revealing an inverse inter-modulation tendency. Elevated Foxp3 protein (by 3.3-fold) was consistently observed in nasal polyps after rapamycin administration by western blot analysis. These findings suggest that rapamycin corrects the dysregulated immune response by enhancing Foxp3 production.

Given that Foxp3 is also expressed by other non-CD4+ T cells, we evaluated the change in the Foxp3+ Treg population in nasal polyps by flow cytometric analysis to further support our hypothesis. We consistently found that the percentage of Foxp3+ Tregs was decreased in nasal polyps compared to the control tissues, confirming the possible role of Foxp3+ Tregs in the immune imbalance of nasal polyps. Moreover, the results for CD4, CD25, and Foxp3 biomarkers in isolated T cells by flow cytometry showed that the frequencies of CD4+CD25+ cells and Foxp3+CD4+ Tregs significantly increased in nasal polyps after rapamycin stimulation. This result is extremely important because it provides direct evidence for Treg expansion in nasal polyps after rapamycin administration.

Furthermore, we examined the changes in T cell cytokines in the supernatants of cultured nasal polyps. A greater level of Th1/Th2 and a lower level of Treg cytokines were detected in nasal polyps compared to controls, confirming that nasal polyps is characterized by mixed types of Th1/Th2 cell infiltration and their corresponding cytokine secretions. After rapamycin stimulation, we observed a significant increase in IL-10 (by 4.4-fold), whereas no significant change in IFN-γ, IL-4, or IL-5 was detected. IL-10 is known as an anti-inflammatory cytokine produced by Tregs and other cells, and is involved in the regulation of immune responses [28]. Therefore, the IL-10 overproduction may be caused by the inhibition of the mTOR signaling pathway, contributing to the balance of the Th1/Th2 network in cultured nasal polyps. However, other cytokines were likely to be unaffected by mTOR signal since they were not significantly changed by rapamycin administration.

Taken together, our results demonstrate that rapamycin is associated with selective Tregs expansion in cultured nasal polyps. During the past few years, the mixed dysregulation of Th1/Th2 in pathogenesis of nasal polyps has been well documented, however, little is known about the importance of Tregs in modulation of the Th1/Th2 imbalance [10,11]. In conjunction with our previous reports, we concluded that the mTOR pathway may play an essential role in the Treg insufficiency, which is characteristic of nasal polyps. Our findings are, therefore, important because they demonstrate the possible association between the mTOR signaling pathway and Foxp3+ Treg expansion in nasal polyps, which may possess clinical potential for developing strategies of treating nasal polyps.

Our study contained some flaws due to technical insufficiency. First, we adopted a tissue culture system since no ideal *in vivo *and *in vitro *model (animal or cellular) for nasal polyps is available at present, thus restricting our experimental plan. Second, it is very difficult for us, as well as other investigators, to take advantage of some reliable methods such as knockdown or mutant constructs to evaluate the importance of the mTOR signal in the development of nasal polyps. Alternatively, we used rapamycin, a specific inhibitor of mTOR, in this study, and provide evidence that blocking the mTOR signal is associated with Foxp3 expression and Foxp3+ Treg expansion in cultured nasal polyps. Our findings suggest that the mTOR signaling pathway may play an important role in Foxp3+ Treg insufficiency and provide some clues about the immunomodulation in nasal polyps. However, the detailed mechanism underlying Treg insufficiency in nasal polyps is still not fully understood and requires further study.

## Conclusion

Our results provide the first evidence that the activation of the mTOR signaling pathway may play an important role in Foxp3+ Tregs insufficiency in nasal polyps and that blocking the mTOR signal with rapamycin is associated with Foxp3+ Treg expansion *in situ*. These findings will be beneficial for better understanding the pathogenesis of nasal polyps, as well as for developing a novel therapeutic strategy for future clinical management.

## Abbreviations

CRS: chronic rhinosinusitis; CRSnNP: chronic rhinosinusitis without nasal polyps; CRSwNP: chronic rhinosinusitis with nasal polyps; Th: T Helper; Treg: T regulatory cell; mTOR: mammalian targets of rapamycin; PI3K: phosphoinositide-3-kinase; RT-PCR: reverse transcription polymerase chain reaction; ELISA: enzyme-linked immunosorbent assay.

## Competing interests

The authors declare that they have no competing interests.

## Authors' contributions

GX and JX performed all laboratory experiments. XH, HZ, CY, ZL, KC and JS helped perform some experiments and analyze the results. HL designed the study, participated in some experiments and prepared the manuscript.
